# Intestinal Obstruction by A Gall-Stone

**Published:** 1909-08-21

**Authors:** 


### Medicine.

### INTESTINAL OBSTRUCTION BY A GALL-STONE.

### SYMPTOMS SUGGESTIVE OF URÆMIA.

Intestinal obstruction by impaction of a large gall-stone within the bowel is a well-recognised, if rare, condition. The following case serves to illustrate the difficulty that may arise in diagnosing not merely the cause of the obstruction, but actually the obstruction itself.

An Instructive Case.

A lady aged 49 came under observation for acute epigastric pain associated with vomiting. It appeared that there had been no previous illnesses of importance, the patient having been perfectly healthy all through an ordinary married life during which she had given birth to three living children and had had one miscarriage. Seven months previous to the acute illness she had had obscure abdominal signs, whose beginning was vague and indefinite; for some while the appetite had been defective and digestion slow and uncomfortable; after each meal there had been a sense of fulness and heaviness, often associated with heartburn and acid eructations.

There had never been any biliary colic, nor even symptoms suggestive of it; no pain in the right shoulder, no jaundice. It was difficult to say even when the slighter abdominal symptoms began to be severe. One night, about six weeks before final admission to hospital, there were acute abdominal pains referred to the epigastrium, accompanied by profuse vomiting, during which, with a sensation of burning heat in the gullet and mouth, the patient averred that she brought up a flood of blood. Nobody else saw this. Next day appetite was fair, and ordinary food was eaten without further hæmatemesis. From this time attacks of vomiting recurred at shortening intervals; at first only some hours after food, but for the four or five days before admission immediately after anything was taken at all.

The patient was a fairly stout woman who was in a state of marked prostration and torpor. She was too languid to answer questions. Vomiting of bile-stained fluid persisted; there had for years past been great constipation, and the bowels had not been opened for three days, although gas was being passed.

The epigastrium was painful, and the pain was increased by pressure, but the parietes were so fat that nothing definite could be felt. The liver did not seem to come below the costal margin, and it was not tender in the least. The urine was scanty, and it contained a moderate quantity of albumen. The heart and the lungs seemed natural. There was no blue line upon the gums. The knee-jerks were present, and there were no Argyll-Robertson pupils. The pulse rate was 96 and the temperature normal. Large enemata were given, and a feeble evacuation of fæcal matter resulted. There was no visible peristalsis, and gas was repeatedly passed per rectum.

The Clinical Course.

Admission having been on the 8th, there was little change to note for some days and the diagnosis was quite obscure. By the 13th the general appearance of the case suggested uræmia. There was complete torpor, with occasional effortless vomiting and occasional periods of sufficient intelligence for the patient to complain of colicky abdominal, chiefly epigastric, pains lasting a few minutes at a time. By the 15th, as the result of further enemata, an abundant motion was obtained. On the 16th, apathy persisting and the cerebral symptoms now being the chief ones to attract notice, lumbar puncture was performed, and some 8 c.cm. of cerebro-spinal fluid removed. There was nothing distinctive about the fluid itself, either chemically or microscopically, except that it contained more urea than usual, a point which seemed to favour the diagnosis of uræmia; but for several hours after its removal there was a decided improvement in the mental condition, the torpor diminishing and voluntary speech being carried on for some time. During that night, however, the vomiting recurred with increased violence, with severe but ill-localised abdominal pains, and the patient died.

Findings at the Autopsy.

At the autopsy the small intestine was found to be enormously distended with gas, and, about a foot above the ileo-cæcal valve two hard bodies were felt fitting tightly within the lumen, though with firm digital pressure they could be pushed onwards. On opening the bowel these two bodies were found to be gall-stones, the smaller measuring 1½ inch in length and 1½ inch in diameter, being behind a larger one 4 inches long and 1 inch in diameter. They were composed almost entirely of cholesterin. The gaseous distension of the bowel began immediately above the site of these gall-stones so that they were clearly the cause of intestinal obstruction.

The gall-bladder was practically non-existent, or rather it was lost in a mass of dense adhesions which bound the under surface of the liver to the duodenum. There was a large fistulous communication between what had formerly been the gall-bladder and the duodenum just beyond the pylorus; clearly it had been by ulceration directly into the duodenum, and not by passage along the cystic and hepatic bile ducts that the gall-stones had found their way into the bowel. The other organs all seemed perfectly healthy, notwithstanding the misleading occurrence of albuminuria during life; the kidneys seemed to be natural both to the naked eye and microscopically.

Some Points of Interest.

Two points at least merit particular attention in the above case. These are:

I. The insidious development and course. During the time the gall-stones were forming there were no symptoms distinctive of biliary mischief. It is most important to realise how much more common gall-stones are than are cases of typical biliary colic. The absence of real colic, and the absence of jaundice, are explained by the fact that the stones never engaged in any of the ducts, but passed directly from gall bladder to duodenum near the pylorus, and this also explains why the pain complained of was in the epigastrium and not over the gall-bladder. During the last phase when the big stones were passing along the intestine the obstruction was never complete enough to make the diagnosis clear: flatus was passed each day, and at intervals sufficient fæcal matter got past the gall-stones to lead to a definite motion coming away per rectum with enemata. It is well enough known that the small intestine is shaped like a very elongated funnel, becoming progressively, though very slowly, narrower from the duodenum above to the ileo-cæcal valve below. Hence it is that gall-stones, when they become impacted in it, are nearly always stuck within three feet of the ileo-cæcal valve.

II. The fact that the oliguria, the albuminuria, and the state of somnolence or torpidity were so striking that an erroneous diagnosis of uræmia was made. The patient was a stout woman of the gall-stone age, and this perhaps should have made the diagnosis easier. Nevertheless it is a striking fact that the great majority of cases of intestinal obstruction by impaction of gall-stones remain undiagnosed except either by exploratory laparotomy, or by autopsy, or by finding the gall-stones ultimately in the fæces.

